# Preeclampsia: From Cellular Wellness to Inappropriate Cell Death, and the Roles of Nutrition

**DOI:** 10.3389/fcell.2021.726513

**Published:** 2021-11-05

**Authors:** Angga Wiratama Lokeswara, Rabbania Hiksas, Rima Irwinda, Noroyono Wibowo

**Affiliations:** ^1^Faculty of Medicine, Dr. Cipto Mangunkusumo Hospital, University of Indonesia, Jakarta, Indonesia; ^2^Maternal Fetal Division, Department of Obstetrics and Gynaecology, Faculty of Medicine, Dr. Cipto Mangunkusumo Hospital, University of Indonesia, Jakarta, Indonesia

**Keywords:** cellular wellness, cell death, apoptosis, autophagy, preeclampsia, nutrition

## Abstract

Preeclampsia is one of the most common obstetrical complications worldwide. The pathomechanism of this disease begins with abnormal placentation in early pregnancy, which is associated with inappropriate decidualization, vasculogenesis, angiogenesis, and spiral artery remodeling, leading to endothelial dysfunction. In these processes, appropriate cellular deaths have been proposed to play a pivotal role, including apoptosis and autophagy. The proper functioning of these physiological cell deaths for placentation depends on the wellbeing of the trophoblasts, affected by the structural and functional integrity of each cellular component including the cell membrane, mitochondria, endoplasmic reticulum, genetics, and epigenetics. This cellular wellness, which includes optimal cellular integrity and function, is heavily influenced by nutritional adequacy. In contrast, nutritional deficiencies may result in the alteration of plasma membrane, mitochondrial dysfunction, endoplasmic reticulum stress, and changes in gene expression, DNA methylation, and miRNA expression, as well as weakened defense against environmental contaminants, hence inducing a series of inappropriate cellular deaths such as abnormal apoptosis and necrosis, and autophagy dysfunction and resulting in abnormal trophoblast invasion. Despite their inherent connection, the currently available studies examined the functions of each organelle, the cellular death mechanisms and the nutrition involved, both physiologically in the placenta and in preeclampsia, separately. Therefore, this review aims to comprehensively discuss the relationship between each organelle in maintaining the physiological cell death mechanisms and the nutrition involved, and the interconnection between the disruptions in the cellular organelles and inappropriate cell death mechanisms, resulting in poor trophoblast invasion and differentiation, as seen in preeclampsia.

## Introduction

Preeclampsia is a multiorgan disorder that is related to pregnancy, characterized by hypertension and endothelial dysfunction (The American Collge of Obstetricians and Gynecologists, 2013). It is one of the leading causes of maternal and fetal mortality and morbidity, with its worldwide incidence estimated to range between 2 and 10% of pregnancies, with higher rate occurring in developing countries (Di Renzo, 2009; Rana et al., 2019). Preeclampsia has long been recognized as a two-stage disease, although latest studies have proposed a five-stage model of the disease with its second and third stage occurring during placentation in early pregnancy (Staff, 2019). This early stage, where decidualization and establishment of maternal–fetal circulation occur, has been the main interest of recent research in search for the best prevention, screening, diagnosis, and therapeutic strategies.

In normal pregnancy, the process of decidualization is of utmost importance as it determines the successful establishment and maintenance of embryo implantation (Okada et al., 2018). The decidualized cells contribute to the microenvironment of endometrium by producing secretory products that influence trophoblast growth and invasion, survival of uterine natural killer cells, immune privileges, antioxidant responses, vasculogenesis, angiogenesis, and spiral artery remodeling (Gellersen and Brosens, 2014).

One of the most important events in the decidua is the series of formation of new blood vessels and vascular alterations, including vasculogenesis, angiogenesis, and spiral artery remodeling, supported by both secretory products of decidua and the extravillous trophoblasts (Cha et al., 2012). In vasculogenesis, *in situ* differentiation of trophoblast takes place, from the totipotent embryonic stem cells into pluripotent hemangiogenic progenitor cells, and eventually into unipotent perivascular cells (Demir et al., 2007). This is then followed by angiogenesis, where new blood vessels arise from the existing ones, including the branched and unbranched vessels through endothelial activation, proliferation and differentiation (Ng et al., 2020). In spiral artery remodeling, apoptotic deaths of maternal endothelial cells and vascular smooth muscle result in transformation of high resistance vessels into low resistance ones, followed by fibrin and endothelial replacement by extravillous trophoblasts (Zhang, 2020). This shows that not only the decidua, but the trophoblasts also play as much role in establishment of appropriate vasoregulation (Cha et al., 2012).

Recent findings have suggested that in order to establish these important vascular changes under hypoxic and stressful environment, trophoblasts need to undergo several physiological cell death mechanisms to support themselves (Saito and Nakashima, 2014). Any disturbances to the cellular health of these cells caused by oxidative stress of any origins may lead to aberrant cellular death mechanisms of the extravillous trophoblast, leading to inadequate spiral artery remodeling and maternal–fetal interface, as seen in preeclampsia. In preeclampsia, inappropriate cellular deaths have been thought to contribute to the abnormal trophoblast invasion. Preeclamptic placenta has been shown to have increased apoptotic and autophagy rate, which causes an alteration of apoptotic cell clearance and further enhances trophoblast cell death (Straszewski-Chavez et al., 2005; Nakashima et al., 2019b). While autophagy and apoptosis are programmed cell death, the excessive rate of which may in turn result in augmented release of syncytial debris (Redman and Sargent, 2007), commonly known as syncytial nuclear aggregates (SNAs), which will enter maternal circulation and activate proinflammatory cytokines and results in endothelial dysfunction seen in preeclampsia (Lau et al., 2013). This systemic endothelial dysfunction manifests as the clinical symptoms of preeclampsia (Redman and Sargent, 2007).

In order to prevent this, appropriate cell death mechanism of the trophoblast must be preserved during placentation. This mechanism, which includes apoptosis and autophagy, requires complex communication and signaling processes, not only between the cells involved, but also intracellularly, inside the trophoblast itself where its organelles function and work in sync (Saito and Nakashima, 2014). Therefore, we propose that cellular wellness, which includes normal cellular integrity and function, is required for trophoblast to maintain its appropriate cell death mechanisms to ensure proper placentation and prevent complications as in preeclampsia, and it can be maintained by adequate nutrition.

The current literature suggests that there is a potential interconnection between the nutritional adequacy, the proper structure and function of each organelle, the cell death mechanisms, the invasion capacity of the trophoblast and preeclampsia (Nakashima et al., 2020). Unfortunately, despite this inherent connection, the currently available studies examined these processes separately and until this review was written, no previous studies and review had provided an in-depth overview of the connection between each cellular structure with regard to their cell death mechanism, nutritional demand, and preeclampsia. Therefore, this review will discuss the relationship of each cellular component of trophoblasts with regard to its cellular death mechanism and preeclampsia, including its cell membrane, mitochondria, endoplasmic reticulum, genetics and epigenetics, as well as environmental contaminants that may potentially affect the cellular wellbeing and, hence, their cellular death mechanism, along with the different roles of nutrients involved.

## Cell Death in Spiral Artery Remodeling

Cell death mechanism is classified into: apoptosis (type I cell death), autophagy (type II cell death), and necrosis/necroptosis (type III cell death). Programmed cell death is a physiological mechanism in order to maintain cellular homeostasis and nutrient cycling. This process includes autophagy and apoptosis. In contrast, unprogrammed cell death such as necrosis and necroptosis (regulated form of necrosis) will cause macrophage damage as well as surrounding cell (Akaishi et al., 2014; Nakashima et al., 2019b). Appropriate trophoblastic cellular death is important in maintaining healthy pregnancy, whereas inappropriate cell death could interfere cellular homeostasis resulting in abnormal placentation leading to preeclampsia.

### Appropriate Cell Death in Normal Pregnancy

There are at least four steps of spiral artery remodeling in pregnancy: (1) decidua-associated remodeling where natural killer cells secrete vascular endothelial growth factor (VEGF) and placental growth factor (PLGF) and angiopoietins, causing the swelling and vacuolation, as well as disorganization of vascular endothelial cells; (2) trophoblastic invasion by interstitial mononuclear cytotrophoblasts occupying an extensive area of uterine wall, followed by intraarterial migration where the invasion of trophoblasts in the spiral arteries eventually leads to temporary replacement of endothelium; (3) further remodeling where the trophoblasts are incorporated into the arterial wall as the penetration of trophoblasts through the smooth muscle cells leads to its replacement by the embedment of the trophoblast in the fibrinoid matrix secreted by the trophoblast itself; and (4) definite reendothelialization occurs as the final step (Sharma, 2017). In these steps, in order to allow for trophoblastic invasion, appropriate cellular death, including autophagy and apoptosis, takes a pivotal role in ensuring a proper establishment and remodeling of the spiral artery.

Autophagy is an important process during differentiation and development including placentation and embryogenesis. Thus, it is crucial in maintaining cellular homeostasis. In a response to hypoxia and oxidative stress, autophagy provides the energy so the cells could overcome starvation by degrading intracellular components. However, this process needs adequate nutrition to be done. Autophagy is regulated *via* activation of mechanistic target of rapamycin complex 1 (mTORC1) by adequate amount of amino acids, glucose, and insulin. This autophagy process primarily occurs in hypoxic condition and hypoxia-reoxygenation in syncytiotrophoblasts (Akaishi et al., 2014; Saito and Nakashima, 2014).

In the beginning of pregnancy, the implantation process of the embryo requires certain phases including apposition, adhesion, and invasion. All of these mechanisms require apoptosis and autophagy. Studies have suggested that blastocysts induce human endometrial cell apoptosis using the Fas/FasL system. Fas, which is present in endometrial epithelium, and FasL, which is present in trophectoderm, enable embryo to breach the epithelial barrier and decidua in the process of implantation (Straszewski-Chavez et al., 2005).

Extravillous trophoblast is a unique trophoblast cell that can invade decidua and remodel uterine blood vessel in order to create an adequate blood supply to the fetus. Studies have suggested that without trophoblast invasion, spiral arteries cannot remodel itself. Moreover, transformation of spiral arteries remodeling requires apoptosis of the endothelial cells, which is induced by the proapoptotic signal from the invading trophoblast. This is shown by the expression of Fas in endothelial cells of the spiral arteries (Straszewski-Chavez et al., 2005). If there is an alteration in proapoptotic signaling from the trophoblast or the response of the endothelium, it may result in lack of vessel transformation (Saito and Nakashima, 2014; Nakashima et al., 2019b).

Villous trophoblast growth is also involved in the physiological apoptotic process. In trophoblast differentiation, cytotrophoblasts will proliferate first, then the fusion of these cells will overlie the syncytiotrophoblast since it does not have the ability to differentiate. Studies have found numbers of apoptotic cascade both in the cytotrophoblast cells and the syncytiotrophoblast. The apoptosis process was thought to be important for cytotrophoblast fusion, formation of syncytial layer, and removal of syncytiotrophoblast aging (Saito and Nakashima, 2014).

There are two types of apoptotic pathways: extrinsic and intrinsic. The extrinsic pathway is initiated by various apoptotic inducing cells such as macrophage and natural killer cells, thus known as death receptor pathways. On the other hand, the intrinsic pathway is initiated as a response to cell injury and DNA damage, known as the mitochondrial pathways (Straszewski-Chavez et al., 2005; Kasture et al., 2021). Apoptosis characteristics, including nuclear condensation, membrane blebbing, and DNA fragmentation, are observed in normal pregnancy, suggesting that the apoptosis process is important in trophoblast growth and remodeling. Trophoblast expresses both death and non-death domain containing TNF-Rs. During stress condition, trophoblast cells will undergo apoptosis, which is shown by the increased activation of Fas and FasL, TNF-R1, and TNF-α, and TRAIL-R1/R2 and TRAIL (Straszewski-Chavez et al., 2005).

### Inappropriate Cell Death in Preeclampsia

In normal pregnancy, placental growth and development are maintained by the increase of apoptotic rate and protective mechanism against senescence by an appropriate level of autophagy (Kasture et al., 2021). However, in pregnancy-related complications like preeclampsia, the excessive rate of trophoblast autophagy and apoptosis occurs, showing that the cell death regulation may contribute to the pathophysiology of the diseases (Kasture et al., 2021).

The presence of highly oxidative stress and hypoxia in preeclampsia is associated with the increase of the autophagic process, particularly in the nutrient-deprived conditions (Dewi et al., 2020). Beclin-1 or Atg6, a protein that enhances autophagic vesicle nucleation and protein recruitment from the cytosol, is found to have a higher expression in the trophoblast and endothelial cells of preeclamptic placenta compared to normal placenta, along with the marker of autophagosome LC3-II. This is followed by a marker of autophagic flux, p62, which is found to be significantly low and inversely correlated with LC3-II, in women with preeclampsia (Akaishi et al., 2014; Dewi et al., 2020). Moreover, there was also differences in autophagy rate in between early and late onset preeclampsia. An *in vivo* study have shown an increase in autophagy, aponecrosis and apoptotic rate in early-onset preeclampsia (Bailey et al., 2017). This was followed by a study in the human placenta, which showed a lower level of LC3B/Beclin-1 ratio in early-onset preeclampsia compared to normal pregnancy, late-onset preeclampsia, and even IUGR (Hutabarat et al., 2017). These findings reflect the failure of trophoblast cell autophagy process in trophoblast differentiation and maturation, suggesting the importance of adequate trophoblastic differentiation since the early stage to enable better cell survival as in normal pregnancy.

Furthermore, inappropriate apoptosis regulation also contributes in the pathogenesis of preeclampsia. A recent study has shown a lower expression of Protein kinase C isoform β (PKCβ), an autophagic regulator, in human preeclamptic placentae compared to normal pregnancy (Zhao et al., 2020). The study showed that a downregulation of PKCβ expression in pregnant mice demonstrates a preeclampsia-like phenotype including hypertension and proteinuria, and is associated with excessive autophagic flux and angiogenesis imbalance. A previous *in vitro* study also showed that suppression of PKCβ promotes upregulation of proangiogenic factor, sFLT1, and downregulation of antiangiogenic factor, VEGFA. These findings also suggested that inappropriate autophagy level might contribute to the impairment of angiogenesis, leading to preeclampsia (Zhao et al., 2020).

Moreover, in preeclampsia, both the extrinsic and intrinsic pathways are activated. The extrinsic pathways of apoptosis is activated by FASL associated with maternal immune system, whereas the intrinsic pathway is stimulated by environmental stress including hypoxia and DNA damage (Raguema et al., 2020). This condition would make alteration *in utero* environment and trophoblast kinetics. As a consequence, the cells would be more susceptible to oxidative damage by triggering more ROS production, the excessive generation of which will recruit more proapoptotic proteins. Preeclampsia is also associated with poor integrin α1β1 expression on extravillous trophoblasts, NK cell dysfunction, and the activation of macrophages, thus increasing EVT apoptosis, necrosis, and tumor growth factor (TGF)-β production by hypoxic stress (Straszewski-Chavez et al., 2005; Akaishi et al., 2014; Saito and Nakashima, 2014). The increase of apoptotic rate in preeclampsia is shown by the increased proapoptotic markers, including p53, Bax, caspase-9, and caspase-3, along with reduction in the antiapoptotic marker Bcl-2 (Straszewski-Chavez et al., 2005). An *in vivo* study also showed that these markers were present in both early- and late-onset preeclampsia (Kasture et al., 2019).

A study using preeclamptic *in vivo* model showed a high-level placental necrosis/necroptosis, compared to normal pregnancy (Denney et al., 2017). Necroptosis is triggered by receptor interacting protein 1 (RIPK1), RIPK3, and mixed lineage kinase domain-like protein (MLKL). The RIP/RIP3 necrosome assembly was induced by the increase of ceramide in preeclamptic placenta, leading to MLKL phosphorylation, and thus necroptosis occurs (Bailey et al., 2017). A study in human placenta also showed a significant increase in RIPK mRNA expression in syncytiotrophoblast (Hannan et al., 2017).

In this vein, the insufficiency of trophoblast invasion and low spiral artery transformation, which is shown in preeclampsia, might be associated with the elevated level of trophoblast cell death. Low remodeling capacity of uteroplacental vessel and placental necrosis are associated with decreased perfusion with low oxygen supply related to cell death (Denney et al., 2017). The mediators of necroptosis also promotes immune response *via* the NF-κB pathway and p38, shown by elevation of cytokine expressions (Zhu et al., 2018). This increase of apoptotic cell level cannot be compensated with adequate macrophage and is aggravated by the pro-inflammatory cytokines, leading to the increase of necrotic trophoblast debris, which could enter maternal circulation. This condition may activate proinflammatory cytokines across systemic circulation, resulting in alteration in vasodilatory endothelial cell function and hypertensive state (Lau et al., 2013). This condition was proved by maternal lactate dehydrogenase (LDH) enzyme, which reflects as a necrosis indicator and has a high negative correlation level with early-onset preeclampsia (Hutabarat et al., 2017).

## Cell Membrane

Within the early pregnancy, the human placenta generates the epithelial trophoblast. The cell membrane of the trophoblast plays a great role in cellular growth, cell migration, and immune response. However, the most important function is to maintain the primary function of placenta, which is maternal-to-fetal nutrient transport to ensure an optimal fetal growth. Trophoblastic cell membrane has apical and basolateral domains. Nutrient transportation across the placenta is initiated from the basal membrane. There are a number of transporters along the membrane, including transporter for amino acids, glucose (GLUTs), and fatty acids (FATPs) for lipid transfer (Lager and Powell, 2012).

The cell membrane, particularly phospholipid, contains polyunsaturated fatty acids that are highly susceptible to lipid peroxidation. This condition may alter the membrane composition, leading to fluidity and permeability changes, ion transport alteration, and metabolic process suppression (Catalá and Díaz, 2016). Bioinformatics analysis in glycosylation and phosphorylation protein process in the placenta suggested that there was a specific cellular process in trophoblastic plasma membrane in patients with preeclampsia (Wang et al., 2013). Studies have found that there was an increase in lipid profile in preeclamptic mother, shown by a high level of triglyceride, LDL-cholesterol, and total cholesterol (Enquobahrie et al., 2004; Ekhator and Ebomoy, 2012). Studies in preeclamptic placenta have suggested that there was an increase in triacylglycerol and cholesteryl ester compared to healthy pregnancy (Brown et al., 2016), as well as an increase of total and individual phospholipid classes (Huang et al., 2013). The elevated lipid profile composition in placenta assembles oxidative stress-induced lipid peroxide environment and thus dysregulates placental syncytiotrophoblast transportation. These conditions are highly associated with maternal endothelial dysfunction (Huang et al., 2013). In addition, this rise of lipid peroxidation may inhibit numbers of cell membrane enzyme such as Na, K-, and Ca-ATPase activities. This condition will lead to the increase of calcium concentration in cytosol in vascular smooth muscle, followed by the increase muscle tension. As a result, there will be a rise in blood pressure, which is a characterization in preeclampsia (Carrera et al., 2003).

### Cell Membrane and Cellular Death

Various lipids such as glycerophospholipids, sphingolipids, and fatty acids, which construct the cell membrane, have been identified to play a role in cell death regulation. The increase of lipid peroxidation, due to imbalance lipid profile in cell membrane, and oxidative stress may trigger death receptors resulting in the increase of apoptosis reaction. In apoptosis, plasma membrane will undergo blebbing, followed by separation and generation of apoptotic bodies (Zhang et al., 2018). However, if there were excessive and aberrant apoptosis cell death mechanisms, they will undergo lytic and inflammatory phases, leading to cell membrane rupture and thus secondary necrosis (Rogers et al., 2017). In addition, the necroptosis process ends up with the loss of integrity of the plasma membrane with MLKL protein as the executor (Zhang et al., 2018, 2020).

Not only the disproportionate apoptotic rate, but a study has also shown that there is a necroptosis process in preeclampsia shown by the increase of ceramide in preeclamptic placenta that enables RIP/RIP3 necrosome assembly. This condition will trigger MLKL phosphorylation. Targeting MLKL in plasma membrane is a crucial process as the oligomerization of MLKL will create a cation channel on the membrane, thus causing high osmotic pressure, water influx, release of intracellular components, and eventual plasma membrane. As a consequence, there will be necroptosis and alteration in cytotrophoblast fusion (Bailey et al., 2017; Zhang et al., 2020).

### Role of Nutrition in Cell Membrane

Studies have been suggesting that dietary component may influence membrane composition, thus altering the activity of membrane-bound enzyme and passive permeability. During pregnancy, alteration in membrane lipid composition placenta might affect transcellular transport such as glucose, water, and amino acids (Powell et al., 1999). Table 1 summarizes the role of nutrients in the maintenance of cell membrane structure and function.

**TABLE 1:** Role of nutrients in the cell membrane.

Nutrients	Role in cell membrane	References
LCPUFA (DHA)	Anti-inflammatory and antioxidant, improving membrane fluidity	Calder, 2016; Burchakov et al., 2017; Godhamgaonkar et al., 2020; Sundaram et al., 2020
Folate (Vitamin B9)	Antioxidant activity and plays a role in mTOR signaling	Williams et al., 2011; Rosario et al., 2017
Vitamin E	Antioxidant activity and modulating protein–membrane interaction	Howard et al., 2011; Zingg, 2015
Choline	Generates phosphatidylcholine and prevents intramembranous material leakage	Penry and Manore, 2008; Godoy-Parejo et al., 2020;
Zinc	Binding site of copper and iron, membrane rheology, cell signaling, enzyme activities, lipid composition maintenance	Verstraeten et al., 2004; Wilson et al., 2017

Fatty acids have an important role in maintaining the structure and function of cell membrane. They regulate membrane ion gates, ion channel receptors, cell signaling, and gene expression, as well as maintain cell architecture and cellular membrane permeability (Duttaroy and Basak, 2020). The most widely known fatty acids that have anti-inflammatory and anti-oxidative properties are long-chain polyunsaturated fatty acids (LCPUFA), particularly the omega 3 derivates. LCPUFA omega 3/omega 6 ratio is important to maintain flexibility and fluidity of phospholipid cell membrane, by acting as immunomodulator *via* lipid-based signaling mediators. Omega 3 counteracts intracellular and membrane damage caused by ROS production such as hydrogen peroxide (Sundaram et al., 2020). Among other omega 3 LCPUFA, DHA is considered to have the most important effect on cell membrane. The DHA content of cell membrane may influence cellular behavior and responsiveness to signals, which may be electrical, chemical, hormonal, or antigenic in nature (Calder, 2016; Godhamgaonkar et al., 2020). The lack of DHA causes the membrane to be less fluid, thus inhibiting a number of important receptors to bind (Burchakov et al., 2017). A previous study found that 800 mg of DHA supplementation in the pregnant obese mother increased fatty acid transporters and reduced placental inflammation (Lager et al., 2017). In addition, DHA may prevent necrosis as it could inhibit TNF-α-mediated necroptosis by interfering with sphingolipid metabolism (Pacheco et al., 2014). Thus, low levels of omega 3, particularly DHA, will allow excessive lipid peroxidation from decreased cell membrane fluidity and overactivation of inflammation. Along with high ROS from the environmental factors, death receptors that enhance trophoblast apoptosis could be activated, which, if it progresses, may lead to necroptosis, resulting in abnormal placentation and vascular remodeling and therefore preeclampsia (Raguema et al., 2020; Zhang et al., 2020).

Folic acid is crucial to healthy pregnancy as it contributes to angiogenesis and vasculogenesis *via* a nitric oxide-dependent mechanism, DNA methylation, antioxidant protection, and endothelial-dependent vascular relaxation. Folate acid has the ability to engulf the free radicals and increase bioavailability NO. A study also found that folic acid supplementation has improved placental development, vascular function, and antioxidant activity in preeclampsia (Williams et al., 2011). Moreover, folate is also found to be a regulator for mTORC1 and mTORC2, which functions to be regulators for amino acid and mitochondria function. As positive signaling for placental function, deficiency of folate is related to alteration of mTOR signaling leading to disruption in nutrient transport (Rosario et al., 2017). Impairment of amino acid transportation across placenta has been highly reported in fetal growth restrictions, which is a common complication found in preeclampsia (Lisonkova and Joseph, 2013; Bahado-Singh et al., 2017).

Choline is a pivotal vitamin-like nutrient that generates one of the abundant phospholipids in the cell membrane, phosphatidylcholine. Deficiency of choline may cause intramembranous material to leak into extracellular fluid as the cell integrity is compromised. It is also involved in synthesis of lipoprotein, acetylcholine, and homocysteine (Penry and Manore, 2008; Godoy-Parejo et al., 2020). Moreover, an *in vivo* study showed that reduction of folate is associated with the increased breakdown of phosphatidylcholine and glycerophosphocholine (GPC), due to a high demand for the methyl group (Chew et al., 2011). This GCP is also found higher in preeclamptic placenta and positively correlates with sFLT1, which is known as a preeclampsia risk factor (King et al., 2017).

Vitamin E is a lipid-soluble anti-oxidant molecule, which plays an important role in modulating protein–membrane interaction in the cell membrane. It works by binding to specific signal transduction involved in phospholipids synthesis, including lipid kinases, phosphatases, and phospholipases. This process also enables vitamin E to prevent lipid peroxidation in the membrane (Howard et al., 2011; Zingg, 2015). A study has found that there was an increase of phospholipids in preeclamptic placenta suggesting the increase of oxidative stress-induced lipid peroxide (Huang et al., 2013). Therefore, a sufficient amount of vitamin E is crucial in maintaining cell membrane integrity by reducing oxidative stress-induced lipid peroxide, which might eventually lead to inappropriate cell death and preeclampsia.

Zinc is important in maintaining plasma membrane structure and function. A study in erythrocyte cell found that zinc deficiency will lead to the increase of osmotic fragility, as well as impairment in platelet adhesion and macrophage function. Cell membrane with zinc deficiency may cause alteration in binding site of other metals such as copper and iron, and affects the membrane rheology, cell signaling, and enzyme activities. Zinc level will also impact the lipid composition in the cell membrane, leading to alteration in lipid properties in a state of deficiency. All of the consequences of a zinc-deficient membrane may cause an increase in lipid oxidation, leading to the generation of oxidative stress condition as the antioxidant properties of zinc, by contributing to Cu/Zn-SOD expression, is defective. The impairment in DNA damage reparation in a state of zinc deficiency also contributes in increasing intracellular oxidative stress. As a result, zinc deficiency causes not only alteration in cell membrane effectivity but also intracellular health defect, leading to the increase of apoptosis *via* internal pathways (Verstraeten et al., 2004; Wilson et al., 2017). The excessive apoptosis rate will then enhance syncytial degradation, releasing inflammatory mediators into maternal circulation. These would promote placentation impairment, leading to endothelial dysfunction as seen in preeclampsia (Ma et al., 2015).

## Mitochondria

Mitochondria play an important role in maintaining placental function as the main site for cellular respiration to meet cellular energy demands. They generate the majority of cellular energy by converting carbohydrates, lipids, and protein into a large amount of adenosine triphosphate (ATP) *via* pyruvate oxidation, fatty acid β-oxidation, the TCA cycle, and oxidative phosphorylation (OXPHOS). Among all of those processes, most of the ATP is produced by the TCA cycle and electron transport chain (ETC). Mitochondria are involved not only in ATP production but also in calcium homeostasis, free radical generation, cell survival, apoptosis, and necrosis (Spinazzola and Zeviani, 2009; Youle and Van Der Bliek, 2012; Pacheco et al., 2014; Marín et al., 2020).

Preeclampsia leads to changes in mitochondrial structure and content, mitochondrial dynamics, as well as apoptosis mechanism (Holland et al., 2017). A study has shown that preeclamptic mitochondria is smaller and has swelling, an irregular arrangement with an incomplete inner and outer membrane, and a decrease in cristae (Zsengellér et al., 2016). Preeclampsia also showed a downregulation in 22 proteins, leading to mitochondrial dysfunction, which is suspected due to long-term hypoxia in placenta (Vishnyakova et al., 2016). This dysfunction leads to impairment in mitochondrial homeostasis. Studies have found that biogenesis has been altered, shown by the low level of TFAM and the high level of OPA1. One study found that preeclampsia is a pro-fusion and anti-fusion environment, shown by the increase in OPA1 and MFN1 and the decrease in FIS1 (Holland et al., 2018). Another study found that there is a decrease in OP1 leading to a pro-fission state (Ausman et al., 2018). Although having a complex pathomechanism, those studies suggested that mitochondrial dysfunction is affected by alteration of the mitochondrial fusion–fission mechanism. In addition, mitophagy has been shown to occur in preeclamptic placenta, shown by an increase in PINK1 gene. This was thought to be caused by an adaptive response of the mitochondria during the increase in oxidative stress (Kalkat et al., 2013).

Alteration in mitochondrial genes affects mitochondrial homeostasis, resulting in mitochondrial dysfunction. This dysfunction leads to the excessive ROS and insufficient ATP production, where they are supposed to generate only 25% ROS during the physiological state (Burton et al., 2017; Holland et al., 2017). In addition, mitochondrial DNA (mtDNA) is also suspected to be the marker of this dysfunction by provoking inflammatory response. Oxidative stress creates membrane potential changes, thus inducing mitochondrial membrane depolarization and increasing permeability. These disruptions will lead to the release of damaged mitochondrial components toward the cytosol, such as ROS and mtDNA. As a result, there will be inflammatory and apoptotic pathway enhancement (McElwain et al., 2020). The damaged mtDNA also alters the ETC mainly *via* complex I and III overproduction. These conditions reflect harmful feedback mechanism, where mtDNA is damaged by oxidative stress, resulting in inefficiency of OXPHOS, causing mtDNA fragment release to the cytosol and, thus, further ROS production as the end product (Lee et al., 2019). This is shown by the increase of toll-like receptor 9 (TLR9), a receptor for mtDNA in initiating pro-inflammatory response, to activate endothelial cells, thus causing endothelial dysfunction in preeclampsia (Marín et al., 2020).

Nevertheless, the increase in ROS generation could be reduced by adequate physiological response to improve cell survival, such as the increased antioxidant defenses, activation of potassium channels, activation of uncoupling proteins, and expression of pro-survival genes (Kalogeris et al., 2014). The free radicals can be counteracted by antioxidants. First-line antioxidant enzymes are SOD, GPx, glutathione reductase, and CAT. Second-line antioxidants are non-enzymatic, low-molecular-weight compounds, such as vitamins C and E, α tocopherol, β-carotene, lipoic acid, ubiquinone, carotenoids, ascorbic acid, uric acid, and glutathione (Birben et al., 2012).

### Mitochondria and Cellular Death

In response to cellular stress such as DNA damage or disturbance of growth factor, p53 will activate proapoptotic Bcl members. P53 works by upregulating death receptor pathways, which triggered the mitochondrial pathways (Straszewski-Chavez et al., 2005). Furthermore, mitochondria can initiate apoptosis by the release of mitochondrial intermembrane space proteins such as cytochrome c into the cytoplasm *via* mitochondrial membrane permeabilization or rupture. This stress-mediated apoptosis is related to cross-talk between mitochondria and ER. In preeclampsia, the mitochondrial apoptosis mechanism seems to be altered as the apoptosis rate is highly far beyond the normal (Bustamante et al., 2014). Studies have found that there is a decrease in proapoptotic proteins such as p53 and BCL2-associated X, and an increase in antiapoptotic protein such as B-cell lymphoma 2 (BCL2) in term preeclamptic syncytiotrophoblast mitochondria compared to the increase of BAX/BLC2 ratio preterm preeclampsia (Holland et al., 2017). In addition, soluble fms-like tyrosine kinase 1 (sFlt-1), which has antiangiogenic activity, was suggested to also play a role in oxidative stress and apoptotic pathways (Shi et al., 2013; Jiang et al., 2015; Zsengellér et al., 2016). Differential apoptosis signaling in preterm and term placenta suggests that mitochondria promote cell survival in placenta by suppressing the apoptosis mechanism. Regulation of programmed cell death and adequate antioxidant activity is important to improve mitochondrial function and adaptation (Holland et al., 2018). In contrast, mitochondrial dysfunction due to excessive ROS production and reduced antioxidant capacity may result in exaggerated apoptotic rate, placentation defect, and therefore preeclampsia.

### Role of Nutrition in Mitochondria

An adequate nutrient availability is important for metabolic pathway regulation and mitochondrial metabolism, thus improving the outcome of clinical imbalance. Healthy mitochondrial function will prevent the increase of apoptotic rate as only a small number of mitochondria are damaged. The nutrients involved in mitochondrial function includes micro- and macronutrients, as summarized in Table 2.

**TABLE 2:** Role of nutrients in mitochondria.

Nutrients	Role in mitochondria	References
Vitamin A	Complexes I and II of ETC	Hebert and Myatt, 2021
Vitamin B1	Improve pyruvate conversion to acetyl-coA	Wesselink et al., 2019; Godoy-Parejo et al., 2020;
Vitamin B2	Complexes I and II building blocks, Fatty acids oxidation in TCA cycle	Wesselink et al., 2019; Godoy-Parejo et al., 2020
Vitamin B3	Precursor of NAD+ for ETC	Wesselink et al., 2019; Godoy-Parejo et al., 2020
Vitamin B5	Precursor of CoA	Wesselink et al., 2019; Godoy-Parejo et al., 2020
Folate (Vitamin B9)	MtDNA biogenesis, antioxidant activity	Rodríguez-Cano et al., 2020
Vitamin B12	Cofactor of succinyl-CoA in TCA cycle	Wesselink et al., 2019; Godoy-Parejo et al., 2020
Vitamin C	Antioxidant activity	Birben et al., 2012
Vitamin D	Promoter of cytokines in mitochondrial fusion, morphology and complexes II–VII of the ETC	Hebert and Myatt, 2021
Vitamin E	Antioxidant activity	Birben et al., 2012
Zinc	Cofactor of antioxidant enzyme (Cu/Zn SOD),	Mistry and Williams, 2011
Copper	Catalytic activity of antioxidant enzyme (Cu/Zn SOD), cytochrome C oxidase enzyme in OXPHOS	Rodríguez-Cano et al., 2020
Selenium	Mitochondrial biogenesis, antioxidant activity	Xu et al., 2016
Iodine	Apoptotic inducers, antioxidant activity	Shrivastava et al., 2006; Cuellar-Rufino et al., 2017; Rodríguez-Cano et al., 2020
Calcium	Apoptotic signaling, TCA cycle inducers	Vishnyakova et al., 2016; Burton et al., 2017

Macronutrients including glucose, lipids, and amino acids pass through mitochondrial metabolism, which results in available metabolites including ATP, acetyl-CoA, NADH, and ROS, where inadequate amount of those substrates may create alteration in the metabolism–epigenome–genome axis. Excessive glucose consumption may cause an increase in ROS production and thus an increase in oxidative stress (Rodríguez-Cano et al., 2020). High-fat maternal diets cause changes in pathways of immune response, inflammation, OXPHOS, and mitochondrial function.

Vitamin A plays a role in improving complex I and II of ETC activity. It is also important to activate PKC-δ, which controls the flux of pyruvate into the TCA cycle. A study has suggested that deficiency of vitamin A may alter embryo reabsorption and poor fetal outcomes (Hebert and Myatt, 2021).

Vitamin B complex plays a significant role in ATP production of the mitochondria. Vitamin B1 is important for pyruvate conversion to acetyl-CoA. Vitamin B2 is essential for complexes I and II building blocks and is involved in the TCA cycle during fatty acid oxidation. Vitamin B3 is the precursor of NAD+ for ETC to work efficiently. Vitamin B5 is the precursor of CoA. Finally, vitamin B12 works as an important cofactor in the formation of succinyl-CoA, the TCA cycle metabolite (Wesselink et al., 2019; Godoy-Parejo et al., 2020). Low levels of folic acid is associated with oxidative damage, altered antioxidant enzymatic activities, decrease in mtDNA biogenesis, and mitochondrial oxidative deterioration. Increased folic acid level also protects mtDNA deletion cumulative damage. An *in vivo* study has found an association between non-supplementation of folic acid, which may reduce hepatic mtDNA, and a higher lipid peroxidation product (Rodríguez-Cano et al., 2020).

Vitamin D plays a role in mitochondria by acting as a promoter of cytokines in mitochondrial fusion, morphology, and ETC, particularly in complexes II and IV. An *in vivo* study also found that calcitriol may reduce apoptosis and oxidative rate in placenta (Hebert and Myatt, 2021).

Antioxidant effectivity is related to functional mitochondrial dynamic process. Vitamins C and E are non-enzymatic antioxidants. Ascorbic acids create intracellular and extracellular aqueous phase antioxidant in order to scavenge oxygen free radicals. Vitamin E is located in the hydrophobic interior site of the cell membrane as it is lipid soluble. It primarily acts to counteract oxidant-induced membrane injury as a ROS scavenger. Vitamin E works by donating electron to peroxy radical, which is produced *via* lipid peroxidation (Birben et al., 2012).

Sufficient amount of zinc may act as a cofactor of antioxidant enzymes, through Cu/Zn SOD pathways. The increased expression of Cu/Zn SOD is one of the intracellular protection mechanisms against free radical damage. It is also important for DNA synthesis by preventing DNA breakdown and promoting DNA repair. It induces cell proliferation, nucleic acid synthesis, carbohydrate and protein metabolism, and cell membrane integrity. However, a high level of zinc could inhibit glycolysis and the TCA cycle (Wesselink et al., 2019). In contrast, zinc deficiency may cause an imbalance between antioxidant and lipid peroxide, resulting in excessive oxidative damage, associated with mitochondrial dysfunction. Placental and maternal serum zinc concentration was also found to be associated with preeclampsia, as the reduction of zinc affects not only antioxidant protection but also the rise in blood pressure (Mistry and Williams, 2011; Ma et al., 2015).

Selenium (Se), a mineral with antioxidant activity as well as a significant trace element, plays its anti-inflammatory and antioxidant role mainly by incorporating into selenoproteins. Selenium is involved in mitochondrial biogenesis by stimulating PGC-1α and NRF-1 (Wesselink et al., 2019). Selenium supplementation has increased mitochondrial respiration and content. Studies have also shown that selenium concentration has an inverse relation with the risk of preeclampsia, which is suspected due to reduction of oxidative stress (Xu et al., 2016).

Copper has a catalytic activity for zinc and SOD. Cu/Zn SOD and manganese SOD are part of the defense antioxidants in the placenta. It also plays a role in mitochondrial OXPHOS, for the effectivity of cytochrome C oxidase enzyme. Low copper is associated with pregnancy-induced hypertension and positively correlated with neonatal weight (Rodríguez-Cano et al., 2020).

Iodine plays a role in trophoblast migration, invasion, and differentiation. It may act as an apoptotic inducer by activating apoptotic cascades *via* mitochondrial pathways, as well as having antioxidant capacity. Studies have shown that iodine deficiency is associated with the rise of oxidative stress and reduction of antioxidant capacity in pregnant women. It also has been associated with pregnancy-induced hypertension in the deficient state (Shrivastava et al., 2006; Cuellar-Rufino et al., 2017; Rodríguez-Cano et al., 2020).

Preeclampsia is associated with intracellular Ca^2+^ alteration. A study showed that there is an increase of mitochondrial respiration in the early onset of preeclampsia. This type of mitochondria is less sensitive to Ca^2+^ depolarization, which reflects mitochondrial adaptation to oxidative stress (Vishnyakova et al., 2016). Ca^2+^ is an important signaling molecule for mitochondria and reticulum endoplasmic interaction for regulating cell death, especially when there is an overload of Ca^2+^. These changes in intracellular Ca^2+^ affect the apoptosis mechanism in placenta (Burton et al., 2017). Calcium-mediated ER signal is able to stimulate the TCA cycle in mitochondria, along with mitochondrial electron transport chain and ATP production to meet protein synthesis requirement (Vishnyakova et al., 2016).

## Endoplasmic Reticulum

As secretory cells, endoplasmic reticulum (ER) plays an imperative role in trophoblasts. ER in a eukaryotic cell functions as the main site of synthesis of secretory proteins and other important factors. During peptide synthesis, ribosomes attach to the rough ER, and nascent peptide chain enters the ER lumen. Inside the lumen, the peptide undergoes spontaneous folding such that the hydrophobic areas are minimally exposed. This folding depends on the availability of energy provided by glucose and disulfide bond formation supported by the oxidizing environment and calcium in the ER lumen. Interestingly, the formation of disulfide bonds is an oxidative event, resulting in significant production of reactive oxygen species (ROS) even under physiological conditions (Geraghty et al., 2015).

### Endoplasmic Reticulum Stress

When there is an imbalance between unfolded protein load and ER folding capacity, ER stress occurs. This will lead to accumulation of unfolded proteins, including the ones necessary for cellular function, such as membrane receptor proteins or hormones. This state of imbalance will then activate a signaling pathway called unfolded protein response (UPR), attempting to restore ER homeostasis (Redman, 2008).

The UPR achieves its aim by reducing the burden through preventing the new polypeptide chain to enter, increasing folding capacity through formation of new ER and more chaperone proteins, and removing accumulated misfolded protein through the ERAD (ER-associated proteasomal degradation) pathway (Redman, 2008). The UPR includes three signaling pathways, which are normally inactivated by the binding of GRP78 to the sensors and is activated by competitive binding of accumulated unfolded proteins. These pathways include PERK (double-stranded RNA-dependent protein kinase (PKR)-like ER kinase), ATF 6 (activating transcription factor 6), and Ire1 (inositol-requiring transmembrane kinase/endonuclease 1) (Guzel et al., 2017). When the UPR fails, apoptotic cascade will then be activated (Redman, 2008).

In preeclampsia, excessive ER stress leads to overexpression of PERK and IRE1α, activating more transcriptional factors ATF4 and ATF6, which would decrease transcription of PIGF, an angiogenic factor required in placentation. Additionally, in preeclampsia, the morphology of the ER also shows that is more dilated and fuller with protein precipitates (Nakashima et al., 2019a).

### Role of Endoplasmic Reticulum in Inappropriate Cell Death

One of the mechanisms controlling cellular homeostasis in placenta is autophagy, through maintaining balance between protein synthesis and degradation. Autophagy promotes protein degradation to prevent buildup of excessive proteins. The protein degradation is achieved either by the autophagy–lysosomal system for long-lived proteins or by the ubiquitin–proteasome system for short-lived proteins (Nakashima et al., 2019a,b). Autophagy may occur both as macroautophagy, a non-selective degradation process where autophagosomes transport internal contents to lysosomes, and as microautophagy, a selective process targeting mitochondria (mitophagy), endoplasmic reticulum (ER-phagy), protein aggregates (aggrephagy), chromatins (chromatophagy), iron-bound ferritin (ferritinophagy), and pathogens (xenophagy) (Nakashima et al., 2019b, 2021).

The relationship between ER stress and poor placentation had been previously established. However, it is important to note that moderate ER stress is in fact, necessary for placental adaptation during the early development (Nakashima et al., 2019b). On the contrary, low ER stress may lead to miscarriage, whereas excessive ER stress may lead to abnormal placental growth as in preeclampsia. When ER stress is excess, chemical inhibitors result in autophagy inhibition, which in turn decreases lysosomes, hence blocking or diminishing autophagy flux (Nakashima et al., 2019a).

Autophagy also has an anti-inflammatory effect by delivering ubiquitinated inflammasome to the autophagosome, whereas when excessive ER stress occurs due to inflammation, non-apoptotic cell deaths occur as the UPR is no longer able to tolerate the accumulated unfolded proteins, including pyroptosis, ferroptosis, and necroptosis. Pyroptosis occurs through the activity caspase-1 on Gasdermin D, IL-1B, and IL-18, whereas necroptosis occurs through serine358 phosphorylation on protein kinases (Nakashima et al., 2020). Additionally, during pyroptosis, autophagy is also inhibited, resulting in secretion of more inflammasomes. Therefore, autophagy seems to be the protective mechanism of trophoblasts against inappropriate cellular death in hypoxic conditions (Nakashima et al., 2019a).

Macroautophagy, ER-phagy, and aggrephagy are reduced or inhibited in preeclampsia due to excessive ER stress, resulting in accumulation of protein aggregates. In aggrephagy, selective autophagy occurs, targeting protein aggregates. These protein aggregates usually consist of misfolded proteins produced by mutations or abnormal translation due to oxidative stress. The aggregated proteins will then be polyubiquitinated and trapped with HDAC6 to form HDAC6 complex. The complex is then transferred to microtubule organizing center (MTOC) *via* dynein, resulting in aggresome, which will attract the autophagosome membrane to form autolysosome together with lysosome. Subsequently, degradation of the inner content occurs by the action of lysosomal hydrolase. This is attributed to the downregulation of transcriptional factor EB (TFEB), which is also retained by the hyperactivation of mTOR in preeclamptic placenta. In preeclampsia, aggrephagy is reduced or inhibited, resulting in more accumulation of protein aggregates (Nakashima et al., 2021).

The ER may also play a role in autophagy through dysregulation of sphingolipids (Melland-Smith et al., 2015). A number of recent studies have also proposed that bioactive sphingolipids are involved in regulating cell death and survival in preeclampsia. Sphingolipids are lipids with sphingoid backbone, including sphingosine, sphingosine-1-phosphate, ceramides, and sphingomyelins (SM). Sphingolipids are important not only as cell membrane constituents with SM as the dominant sphingolipids in mammalian cells, but also as signaling pathway molecules (Slotte, 2013). Although found in cell membrane, sphingolipids are synthesized by endoplasmic reticulum, and found to be increased in preeclampsia. Among these sphingolipids, ceramides are thought to be the important signaling molecule in cellular response to stress and cell death. Ceramide levels are influenced by its *de novo* synthesis in the endoplasmic reticulum, the catabolism of sphingomyelin, and its breakdown into sphingosine. Long-chain ceramides (C16–C24) are thought to be important inducers of autophagy, whereas sphingosine-1-phosphate (S1P, a ceramide metabolite in the breakdown of ceramide) is thought to promote cell survival.

A recent study has suggested that the oxidative stress environment in preeclampsia causes an increase in *de novo* synthesis of ceramides, resulting in increased autophagy in trophoblast, by activating the MAPK8/JNK1 pathway which frees Beclin-1 to initiate autophagy. Interestingly, this study also found that the increase in ceramide was not due to increased breakdown of sphingomyelin (shown by reduced activity of the enzyme SMPD1), but rather due to the increased *de novo* synthesis as part of the response to oxidative stress (Melland-Smith et al., 2015). Another study investigating the alterations of sphingolipids in endothelial cell of chorionic artery has also suggested increased sphingomyelins and ceramides, and reduced S1P, all of which point toward endothelial dysfunction (Del Gaudio et al., 2020).

### Role of Nutrition in Endoplasmic Reticulum

Endoplasmic reticulum stress may be induced by some stimuli, including accumulation of unfolded proteins, fatty acids, cytokines, redox state dysregulation, or increased intracellular calcium (Flamment et al., 2012). Table 3 summarizes the role of nutrients in the maintenance of ER structure and function.

**TABLE 3:** Role of nutrients in endoplasmic reticulum.

Nutrients	Role in endoplasmic reticulum	References
Fatty acid	Inducer of ER stress	Colvin et al., 2017
Calcium	Maintenance of oxidizing environment for disulfide bond formation in protein folding	Deniaud et al., 2008; Mekahli et al., 2011
Glucose	ATP for protein folding	Geraghty et al., 2015
Obesity	Protein misfolding and ER stress	Flamment et al., 2012; Liong and Lappas, 2015
Vitamin C	Reducing ER stress induced by high glucose	Yung et al., 2016
Vitamin D	Suppression of ER stress	Riek et al., 2012; Haas et al., 2016;
Vitamin E	Reducing ER stress induced by high glucose	Yung et al., 2016

Fatty acids are one of the known inducers of ER stress. However, not all fatty acids affect the ER stress similarly. Unsaturated fatty acids, the highest concentrations in most diets of which is palmitate, are found to be able to induce ER stress, causing lipotoxicity in trophoblasts through the caspase-mediated pathway. In contrast, several dietary unsaturated fats such as oleate, linoleate, eicosapentaenoic acid, and docosahexaenoic acid have lipoprotective activities through the formation of neutral lipid triglycerides, hence preventing formation of diacylglycerol, which may trigger ER stress and cellular damage. Therefore, the maternal ratio of saturated compared to unsaturated fatty acids may be an imperative factor in determining ER stress-mediated placental dysfunction (Colvin et al., 2017).

Previous studies have shown that ER stress is increased in obese pregnant women and women with gestational diabetes mellitus. This is not surprising as ER stress plays a central role in the insulin resistance in obesity and type 2 diabetes in general. In these obese women, the adipose tissue results in higher inflammasome and secretes cytokines, which may cause insulin resistance, where ER stress is a prominent feature (Liong and Lappas, 2015). In obese state, there is a decrease in the enzyme responsible for disulfide bond formation in the ER (Dsba-L), hence causing misfolding and failure in multimerization of adiponectin, subsequently leading to ER stress (Flamment et al., 2012). In terms of diet, traditionally, obesity is associated with excessive caloric intake, which consists of carbohydrates, especially simple ones, and fats. Additionally, recent studies have also shown that obesity is also associated with micronutrient deficiencies such as vitamins D, A, B, and E; potassium; calcium; folic acid; iron; zinc; iodine; selenium; and manganese.

Calcium ion is important not only in the cytoplasm but also inside the ER. As a place of calcium storage, at least three different types of proteins must be expressed in the ER: calcium ion pumps for transport of calcium ion from cytosol to ER lumen against electrochemical gradient such as the SERCA (sarco/endoplasmic reticulum Ca^2+–^ATPase); luminal calcium ion-binding proteins such as calsequestrin, calreticulin, or calnexin to store the calcium ion; and calcium ion channel for the controlled release of calcium from the ER to the cytosol in line with its electrochemical gradient such as the RyR (ryanodine receptor) and IP3R (inositol triphosphate receptor). In physiological conditions, several mechanisms will compensate for release of calcium from the ER by increasing mitochondrial activity to increase ATP and SERCA activity and calcium influx to restore the calcium inside the ER. On the other hand, depletion of calcium inside the ER may trigger ER stress through impairment of the normal mechanisms and the calcium channels. ER calcium depletion occurs in many chronic metabolic diseases such as diabetes, neurodegenerative diseases, and cardiovascular diseases (Mekahli et al., 2011). Calcium homeostasis in the ER is also important in the process of apoptosis. During ER stress, more calcium is being transported out of the ER into the cytosol. This creates a high concentration of calcium inside the cytosol, which when protracted above its critical threshold, induces proapoptotic mitochondrial membrane permeabilization (Deniaud et al., 2008).

Additionally, other previous studies on some vitamins have also shown to affect the ER stress pathways, namely, vitamins C, D, and E. A study by Haas et al. (2016) showed that vitamin D inhibits ER stress in endothelial cells whereas another study by Riek et al. (2012) showed that vitamin D is able to suppress ER stress in monocytes of type 2 diabetes patients. A study on gestational diabetes patients showed that addition of vitamins C and E results in reduction of placental ER stress induced by high glucose by restoring its pH, although the exact mechanism of which remains unkown (Yung et al., 2016).

## Genetics and Epigenetics

The development of the placenta is a complex process, both functionally and genetically. The maternal–fetal interface has to incorporate two semiallogenic individuals, the fetus and the mother. In order to accommodate both interests, placenta is made up of different cells with differing structures and functions, and consequently different sets of gene expression. A single-cell transcriptome study has now revealed the different gene expression profiles in different cells of the placenta. Extravillous trophoblast cells have a unique expression profile with the expressions of non-classical human leukocyte antigen (HLA-G) and integrin 5 alpha (ITGA5), KRT7, and ADAM12 (Pavličev et al., 2017). These cells also differ from cytotrophoblast by the expression of some endothelial genes, including MCAM, which may contribute to cell motility, needed especially for invasion in early pregnancy (Ye et al., 2013). On the other hand, maternal uterine dendritic cells and decidual cells express different sets of genes, depending on their functions. These cells communicate with each other through ligand-receptor binding, and these interactions change during decidualization, where growth factors such as FGF9, BMP2, and PDGFB are upregulated (Pavličev et al., 2017).

As an organ with multifaceted functions, it is not surprising that complex epigenetic mechanism plays an imperative role in the development of placenta. Through DNA methylation, chromatin remodeling, and histone modifications, tissue-specific gene expression is regulated at the transcription level, whereas non-coding RNAs, including miRNAs, are regulated at the posttranscriptional level (Gundacker et al., 2016).

In the context of pregnancy, placenta is hypomethylated compared to the embryo, especially in some regions of the X chromosome. Some known hypomethylated regions in the placenta include long interspersed elements-1 (LINE-1) and AluYb8 (Wilhelm-Benartzi et al., 2012). Despite being generally hypomethylated, some regions in the placenta are actually hypermethylated. These include tumor suppressor genes such as Ras association domain family 1 isoform A, and oncogene Wnt2 (Januar et al., 2015). Various published animal studies have shown the significance of DNA methylation to placental function. In mice, DNMT3L knockout leads to trophoblast giant cell proliferations (Arima et al., 2006). Treating pregnant rats with demethylating agent has also led to decreased placental weight, and abnormal trophoblast proliferation (Serman and Dodig, 2011). Emerging evidence has shown that abnormal placental epigenetics leads to abnormal placental functions (Januar et al., 2015).

Non-coding miRNAs also help downregulate gene expression post-transcriptionally by binding to complementary mRNAs. In the placenta, for example, miRNA-328 has been known to influence expression of BCRP (breast cancer resistance protein) (Gundacker et al., 2016). Other miRNA known to be highly expressed in the placenta include miR-141, miR-149, miR299-5p, and miR-135b (Maccani and Marsit, 2009).

Genetic imprinting has also been proposed to be a part of epigenetic reprogramming during germ-cell development and maintained through the preimplantation development. Genetic imprinting determines expression of genes in a mono-allelic, parent-specific pattern. As a maternal–fetal interface, placenta becomes a place for both paternal and maternal genes. Therefore, many imprinted genes are exclusively imprinted in the placenta (Gundacker et al., 2016). Previous research in mice has shown that deletion of the paternally expressed gene (Igf2, Peg1, and Peg3) results in smaller placenta, whereas deletion of the maternally expressed gene (H19, Igf2 receptor, Cdkn1c, Phlda2, and Grb10) results in larger placenta, suggesting that paternally imprinted genes are growth promoters while maternally imprinted genes are growth suppressors (Constância et al., 2005).

### Genetic and Epigenetic Changes in Preeclampsia

#### Gene Expression

At present, previous research has shown that there is a strong familial predisposition of preeclampsia. The risk of having preeclampsia is increased five and two times in women with first-degree and second-degree relatives with preeclampsia, respectively. It is also worth noting that genomic imprinting results in paternal genes playing significant roles in the invasion and placental growth and maternal genes playing significant roles in the adaptive immune response. Several studies have connected the roles of some single-nucleotide polymorphisms (SNPs) on the genes involved in preeclampsia through the process of immune maladaptation, vascular and endothelial function, thrombophilia disorders, and metabolism and oxidative stress. These genes include the following: (Valenzuela et al., 2012; Michita et al., 2018; Wang and Lian, 2019).

(1) Immune maladaptation: ERAP1, ERAP2, TNFSF13B, and HLA-G Gene Polymorphism.
(2) Vascular and endothelial function: VEGF, eNOS, and CYP11B2.
(3) Thrombophilia disorders: Human prothrombin F2, Factor V Gene, and SERPINE1 GENE.
(4) Metabolism and oxidative stress: PON-1, MTHFR, MTRR, and MTR Genetic Polymorphism.
(5) Apoptosis: TNFSF13B, Fas, FasL, and MBL2.

Another review has proposed a set of candidate genes and polymorphisms that are known to play some roles in preeclampsia and the HELLP syndrome. This includes STOX1 gene, syncytin envelope gene, MBL gene polymorphism, Factor V Leiden mutation, MTHFR polymorphism, G021A mutation in the Factor II (prothrombin gene), and VEGF (Haram et al., 2014).

In preeclampsia, alterations in methylation may also affect trophoblast invasion through the expression of MMP genes. MMP consists of several Zn^2+^- and Ca^2+^-dependent proteases that degrade the extracellular matrix. MMP-2 and MMP-9 reduce the remodeling of spiral arteries in early gestation. A hypomethylation of TIMP3, an inhibitor of MMP, has also been found in preeclampsia, causing increased transcription of TIMP3, hence inhibiting angiogenesis and vascular endothelial growth factors (Apicella et al., 2019).

#### Aneuploidy

Another possible genetic abnormality found in preeclampsia is the confined placental mosaicism (CPM) where there is a mosaic trisomy confined to the placenta. This condition is found when chromosomal non-disjunction occurs post zygotic stage, more specifically after the separation of fetal and placental compartments. CPM is more common than non-mosaic trisomy as most non-mosaic trisomies (with the exception of Trisomy 13, 18, and 21) will lead to spontaneous abortions. Trisomy in placenta may adversely affect differentiation of cytotrophoblast. CPM is divided into three subtypes according to the cells affected: Type I is where the CPM is confined to the cytotrophoblast, Type II is where the CPM is confined to mesenchymal core, and Type III is when the CPM occurs in both. Studies have shown that some CPM are associated with preeclampsia, namely, CPM of trisomy 14 and trisomy 16. The former may be attributed to the fact that FLT1 gene is localized on chromosome 13 (Jebbink et al., 2012; Gundacker et al., 2016).

#### Epigenetics

Previous research has proposed the concept of epigenetic drift, where environmental factors such as nutrition, chemicals, and temperature changes may influence gene regulation. This is particularly apparent in pregnancy where there are developing placenta and fetus. New evidences have proposed that dysregulation of placental epigenetics may contribute in placental dysfunction (Gundacker et al., 2016).

In preeclampsia, dysregulations of methylations have been found. Interestingly, these alterations are different between early and late onset of preeclampsia, suggesting possible different etiology. In early-onset preeclampsia, there were more global or genome-wide hypermethylation, compared to late-onset preeclampsia, probably because epigenetic reprogramming occurs due to earlier stress. The known hypermethylated genes in preeclampsia include WNT2 and IGF-1 genes for placentation and cell signaling, GATA4 for placenta growth, CDH11 for trophoblast anchoring and syncytiotrophoblast differentiation, and HLA-G gene for maternal immune tolerance and rejection. This high global methylation of preeclampsia may be associated with increased homocysteine level. Nevertheless, in preeclamptic state, some genes are also hypomethylated, including INHBA and BHLNE40 genes for inhibition of trophoblast differentiation (Apicella et al., 2019).

Moreover, several studies have also explored miRNA in preeclampsia. There were several miRNAs found to be upregulated in preeclampsia, among them are miR-210, miR-155, and miR-200b. miR-210 is associated with regulation of mitochondrial function and hypoxia response, whereby under hypoxic conditions, it is able to switch mitochondrial function to promote glycolysis. An *in vitro* study also showed that an overexpression of miR-210 leads to downregulation of invasion and migration. On the other hand, miR-155 regulates trophoblast proliferation by downregulating Cyclin D1 cell cycle gene, causing cell cycle arrest, and decreased proliferation (Pineles et al., 2007; Apicella et al., 2019).

### Role of Genetics and Epigenetics in Cellular Death

The autophagy as a cellular death mechanism is controlled both transcriptionally and epigenetically. At the transcriptional level, the expression of genes causing enhanced autophagy is controlled by transcription factors ATF4, CHOP, FOXO1, HIF1, p53, and many others (Füllgrabe et al., 2014). A tumor suppressor gene p53 inhibits G1-to-M phase transition in cell cycle when exposed to glucose starvation in order to ensure cell survival. Under this stress, the activated p53 also activates other transcription factors that are responsible for autophagy, including Sestrin 1/2, TSC2, and 5′ adenosine monophosphate-activated protein kinase (AMPK) β1/β2 (Gong and Kim, 2014), whereas the expression of genes causing suppressed autophagy is controlled by transcription factors ATF5, β-catenin, STAT1, STAT3, and others (Füllgrabe et al., 2014). Epigenetically, there are several miRNAs controlling genes associated with autophagy, which include RB1CC1, ULK1, ULK2, ATG4, ATG9, and others, along with H3 histone modifications (Füllgrabe et al., 2014).

In physiological conditions, some of the other known epigenetics modifications also occur at the genes regulating for cellular deaths such as apoptosis. These involve another set of non-coding RNA aside from mRNA, which is the long non-coding (lnc) RNA, namely, lncRNA MALAT1, lncRNA MEG3, lncRNA SPRY4-IT1, and lncRNA H19 (Apicella et al., 2019).

Genetic imprinting seems to also play a role in the regulation of cellular deaths, autophagy, and apoptosis. The aplasia Ras homolog member I (ARHI) is known to be maternally imprinted, and in an *in vitro* study of placenta with only paternal genes, autophagy is increased. On the other hand, the PHLDA2 gene is known to be paternally imprinted, and in an *in vitro* study of placenta with only maternal genes, the gene is overexpressed, inducing apoptosis through caspase cascade activation (Ptak et al., 2014).

On the other hand, preeclamptic placenta has been shown to have increased in apoptotic and autophagy rate, which cause an alteration of apoptotic cell clearance and further enhancing trophoblast cell death (Straszewski-Chavez et al., 2005; Nakashima et al., 2019b). In preeclampsia, some differentially hypomethylated genes in preeclampsia are also known to be associated with the increased activities of the TGF-β pathway. These genes include SMAD3, SKI, RUNX3, and TGIF1. TGF-β is known to be increased in preeclamptic patients as inhibitors of angiogenic factors (Martin et al., 2015).

In preeclampsia, some alterations in methylation and miRNA have also been found. The differentially methylated genes include hypomethylation of P2RX4 gene and FN1 gene, which results in abnormalities in the apoptosis, trophoblast proliferation, and differentiation. Additionally, dysregulated miR-183 and miR-21 have also been found to affect apoptosis and trophoblast invasion (Apicella et al., 2019).

### Role of Nutrition in Gene Expression

Table 4 summarizes the role of nutrients in gene expression and epigenetics. Folate is a B vitamin that is essential for the synthesis of purine and thymidine nucleotides required for DNA replication and repair, as well as for production of methionine from homocysteine in one-carbon metabolism cycle for the purpose of methylation reactions. After being transported into the cells in its monoglutamate form and processed into functional tetrahydrofolate polyglutamates inside the cell, folate in the form of tetrahydrofolate will be converted into 10-formyltetrahydrofolate for the production of purines, requiring ATP in the process and some into methylenetetrahydrofolate for the production of thymidine, requiring serine amino acid in the process. Aside from ATP and serine, it is also important to note that one-carbon metabolism can also be affected by the deficiency in vitamin B12, iron, and riboflavin (Ducker and Rabinowitz, 2017).

**TABLE 4:** Role of nutrients in genetics and epigenetics.

Nutrients	Role in genetics and epigenetics	References
Folate	• Synthesis of purine and thymidine nucleotides required for DNA replication and repair.	Ducker and Rabinowitz, 2017
	• Methyl donor in one-carbon cycle for DNA methylation.	
Zinc	• Stabilizing the structure of DNA, RNA, and ribosomes.	Beyersmann and Haase, 2001; Klug, 2010
	• Formation of zinc finger domains and cofactor of RNA polymerases and DNA polymerases.	
	• Incorporates thymidine in cell cycle.	
Choline	Methyl donor for DNA methylation	Choi and Friso, 2010
Betaine	Methyl donor for DNA methylation	Tiffon, 2018
Vitamin A	• DNA methylation.	Huang et al., 2018
	• Histone modification.	
	• miRNA regulation.	
Vitamin B1	Histone modification	Huang et al., 2018
Vitamin B2	• Participates in one-carbon cycle for DNA methylation.	Geraghty et al., 2015; Huang et al., 2018
	• Histone modification.	
	• miRNA regulation.	
Vitamin B3	• Participates in one-carbon cycle for DNA methylation.	Geraghty et al., 2015; Ducker and Rabinowitz, 2017; Huang et al., 2018
	• Histone modification.	
Vitamin B6	Cofactor in transsulfuration pathway producing antioxidants	Kasture et al., 2018
Vitamin B7	DNA methylation	Huang et al., 2018
Vitamin B12	• Participates in one-carbon cycle for DNA methylation.	Geraghty et al., 2015; Ducker and Rabinowitz, 2017; Huang et al., 2018
	• Histone modification.	
	• miRNA regulation.	
Vitamin C	• DNA methylation.	Huang et al., 2018
	• Histone modification.	
	• miRNA regulation.	
Vitamin D	• DNA methylation.	Huang et al., 2018
	• Histone modification.	
	• miRNA regulation.	
Vitamin E	• DNA methylation.	Huang et al., 2018
	• Histone modification.	
Vitamin K	• DNA methylation.	Huang et al., 2018
	• miRNA regulation.	
DHA	• Transcription of genes involved in lipid metabolism.	Rosa Velazquez et al., 2020
	• DNA methyltransferase (an enzyme required for DNA methylation).	
High-fat diet	Hypermethylation of certain promoter genes	Masuyama et al., 2015

Many previous studies have suggested the role of zinc in the regulation of DNA synthesis and mitosis. Zinc is known to play a role in stabilizing the structure of DNA, RNA, and ribosomes, and is hence present in the cell nucleus, even in nucleolus. Zinc acts as a necessary cofactor of the enzymes required in DNA replication and translation, including RNA polymerases (Powell, 2000; Beyersmann and Haase, 2001; Klug, 2010). In these enzymes, zinc forms a zinc finger domain (where zinc ion forms a loop in the polypeptide chain and creates a bridge between cysteine and histidine residues). These zinc finger domains are considered necessary for the binding of these enzymes to the DNA sequences for transcription to occur. Therefore, zinc plays an important role in gene expression and regulation (Klug, 2010). Furthermore, animal studies have also shown that a zinc-deficient diet in rats causes a linear decrease in thymidine incorporation into DNA, which is required for G1 and early S phase of cell cycle where DNA replication occurs. Zinc is also known to be essential in the stimulation of IGF-1, which is also important in cell cycle (Powell, 2000; Guo et al., 2020).

### Role of Nutrition in Epigenetics of Preeclampsia

During fetal development, the epigenetic alterations are much more likely to occur under environmental stimuli, one of which is nutrition. Some nutrients may influence DNA methylation through their interaction with the one-carbon metabolism cycle, a cycle producing methyl groups. These methyl donor nutrients include folate, vitamin B12, vitamin B6, vitamin B2, choline, and betaine. In one carbon cycle, folate plays a pivotal role as it donates one carbon in the form of 5-methyl-tetrahydrofolate, hence causing remethylation of homocysteine to methionine catalyzed by methionine synthase, which is vitamin B12-dependent (Geraghty et al., 2015). Alterations in the one carbon cycle may lead to depleted supply of methyl group, hence affecting DNA and histone methylation and gene expression, whereas alterations in the transsulfuration pathway may lead to elevated levels of homocysteine and ROS, resulting in oxidative stress, endothelial dysfunction, and upregulation of apoptotic markers (Kasture et al., 2018).

A number of animal studies have previously been published investigating the effect of the aforementioned methyl donors in maternal diet and supplementation to offspring DNA methylation. In these studies, folic acid is able to prevent hypermethylation of IGF2 and H19 genes, and prevent hypomethylation of GR and PPAR alpha genes (Sinclair et al., 2007). One study has also shown that choline deficiency would result in global and IGF2 hypermethylation (Kovacheva et al., 2007). Additionally, another study published that a high-fat diet leads to hypermethylation of H3K9 histones (adiponectin) in the promoter region of leptin gene (Masuyama et al., 2015). On the other hand, in human interventional and observational studies, folic acid supplementation is shown to have no significant association with global methylation but is associated with lower methylation on DNA sequences deregulating IGF2 expression, and lower methylation at the ZFP57 gene (Fryer et al., 2009; Amarasekera et al., 2014). With regard to other methyl donors, high maternal vitamin B12 was associated with lower global DNA methylation, there was a positive correlation of dietary vitamin B2 and ZAC1 methylation, and betaine and choline were inversely associated with global methylation in males (Azzi et al., 2013).

Choline is also considered to be one of the methyl donors in one carbon cycle. Previous studies have shown that choline deficiency leads to either reduced or increased methylation of histone especially in genes regulating neurogenesis and hippocampal development (Choi and Friso, 2010). Associated with choline, betaine is also considered to be a methyl donor and hence influences DNA methylation (Tiffon, 2018).

Aside from folate, a few other vitamins are also known to assert effects on epigenetic modifications. DNA methylation can be affected by vitamins A, B2, B3, B7, B9, B12, C, D, E, and K; histone modification can be affected by vitamins A, B1, B2, B3, B9, B12, C, D, and E; and non-coding RNA expression may be affected by vitamins A, B2, B9, B12, C, D, and K. The detailed functions of those vitamins in relation to epigenetic regulations can be found in a study by Huang et al. (2018).

DHA, or omega 3 PUFA, has also been known to play a role in regulation of gene expression. It affects gene transcription and enzymes of DNA methylation [DNA-methyltransferase (DNMT)-3A], especially the ones involving lipid metabolism, although much of the available studies currently are restricted to animal studies (Rosa Velazquez et al., 2020).

## Environmental Factors

Recent studies have also highlighted the importance of a group of substance termed endocrine disrupting chemicals (EDCs). These EDCs are mostly man-made and include organochlorine pesticides (O), plastics [e.g., bisphenol A (BPA)], cosmetic products, plasticizers [e.g., phthalates (PAEs)], and pharmaceutical agents [e.g., diethylstilbestrol (DES)], parabens, polychlorinated biphenyls (PCBs), perfluorinated compounds (PFCs), organophosphates, dichlorodiphenyltrichloroethane (DDTs), polybrominated diphenyl ether (PBDE), and organotins (Tang et al., 2020). Exposure to EDCs during pregnancy have recently gained research interest as they may pose a long-term effect on developing fetus. This is especially due to the abundance of hormone receptors in the placenta, which makes the placenta and fetus particularly vulnerable to the effects of EDCs. These chemicals have been suggested to interfere with trophoblast invasion and spiral artery remodeling, which could potentially result in placental pathologies such as preeclampsia (Gingrich et al., 2020). A Chinese birth cohort found positive association between PFCs found in umbilical cord blood and preeclampsia (Huang et al., 2019).

Some of these compounds are found to be affecting cell death regulation in the placenta, by inducing apoptosis of trophoblast cells, and placental necrosis. The EDCs that have been suggested to alter cell death regulation include PCBs, PFCs, parabens, phthalates, and bisphenols. A comprehensive review detailing the effect of each compound can be found in Gingrich et al. (2020). Some of the studies that are highlighted include an *in vitro* study where parabens inhibit cell proliferation and induce apoptosis and ER stress (Yang et al., 2018), another *in vitro* study where PCB mixtures are shown to induce apoptosis of the trophoblast cell (Gu et al., 2012), and a study where PFC was shown to decrease cell viability and induce apoptosis (Zhang et al., 2015).

Environmental contaminants may also have damaging effects on the trophoblasts, leading to poor placentation and preeclampsia. Potential contaminants affecting placental health that have been previously studied and reviewed include persistent organic pollutants (POP), drinking water contaminants, air pollutants (ozone, particulate matter PM10 and PM2.5, carbon monoxide, lead, sulfur dioxide, and nitrogen dioxide), and metals (arsenic, cadmium, chromium, and mercury) (Rosen et al., 2018).

Persistent organic pollutants (POP) are organic chemical substances used for agricultural and industrial purposes, such as in pesticides (dichlorodiphenyltrichloroethane or DDT and hexachlorocyclohexane or HCH), industrial chemicals (hexachlorobenzene or PCB and polybrominated diphenyl ether or PBDE), and their by-products (PCB, dioxins, and furans) which persist in the environment for years. Contradicting results have been published regarding the association between PCB and preeclampsia, and a review by Rosen et al. (2018) suggested that no conclusion can be drawn just yet (Savitz et al., 2014; Eslami et al., 2016). Similarly, previous studies have also shown no clear correlation between DDT exposure and preeclampsia (Savitz et al., 2014).

In terms of water contaminant, tetrachloroethylene (PCE) and trichloroethylene (TCE) show association with an increase in pro-inflammatory cytokines leading to trophoblast dysfunctions *in vitro*. Nevertheless, the concentration used in this experiment was higher than acceptable maximum standard in the United States, and one observational study involving 1,766 women proves otherwise. Therefore, no conclusion regarding the role of water contaminant on the incidence of preeclampsia can be drawn (Chen, 2004; Carwile et al., 2014; Hassan et al., 2016).

In contrast, previous studies have supported the association between air pollutants and preeclampsia. A meta-analysis of 16 studies by Pedersen et al. (2014) found significant associations between PM2.5, NO2, and traffic with preeclampsia. This finding is also supported by other studies in Danish National Cohort and in China, though some studies did not agree with this finding (Klemmensen et al., 2007; Pedersen et al., 2014; Wang et al., 2018). Genetically, exposure to PM2.5 is found to lower the degree of placental global DNA methylation as well as mitochondrial methylation (Janssen et al., 2013).

Heavy metals such as cadmium, chromium, and mercury are naturally occurring substances but are often found in products for industrial, domestic, and agricultural uses. This includes arsenic, cadmium, chromium, and mercury. In general, studies investigating the impacts of heavy metals on preeclampsia have been limited. However, some of them have found association between them (Rosen et al., 2018).

## Integrated Cellular Death Mechanism and Nutrition Involved

**FIGURE 1:**
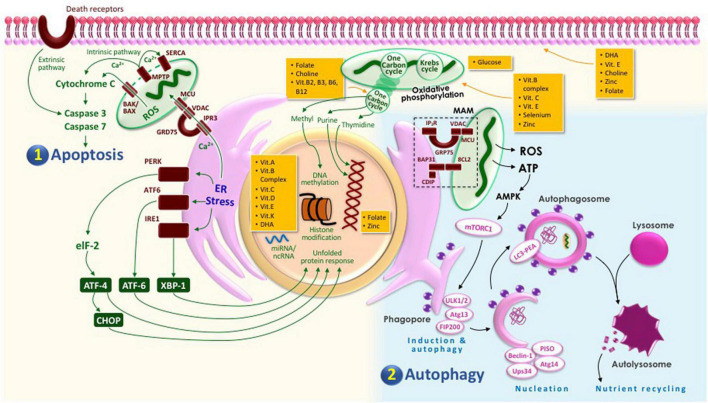
Physiological cell death pathways in a trophoblast cell and the associated nutrition required in maintaining cellular wellness. In a physiological state, the following cell death mechanisms occur in a trophoblast in order to ensure appropriate invasion: (1) apoptosis and (2) autophagy.

Figure 1 summarizes the physiological cell death pathways in a trophoblast cell and the associated nutrition required in maintaining cellular wellness. In a physiological state, the following cell death mechanisms occur in a trophoblast in order to ensure appropriate invasion: (1) apoptosis and (2) autophagy. Apoptosis may be activated through either extrinsic pathway or intrinsic pathway. In extrinsic pathways, activated death receptors (Fas, TRAIL, and TNF) will trigger caspase 3-mediated cascade, leading to apoptosis, whereas, in intrinsic pathway, endoplasmic reticulum (ER) stress will induce calcium efflux from the ER *via* IP3R channel, whereby some of the calcium will be transported back into the ER *via* SERCA channel, and others will then enter the mitochondria through VDAC and MCU channels in the mitochondria-associated membrane (MAM) compartment, where there is direct contact between ER and mitochondria. Calcium influx into the mitochondria will trigger release of cytochrome c *via* MPTP channel leading to apoptosis. It is also worth noting that moderate ER stress is required in order to activate the UPR to ensure protein homeostasis. On the other hand, autophagy is triggered by the hypoxic condition in the maternal–fetal interface, leading to less ATP being produced by the mitochondria, leading to more AMPK and, hence, less mTORC1, which will then result in free ULK complex. Together with Atg13 and FIP200, the ULK1/2 will form a complex, inducing the formation of phagophore, which will be nucleated by the Beclin-1–P150–Vps34–Atg14 complex. The elongation process will follow, involving Atg complexes, leading to formation of LC3-PEA, which will recognize ubiquitinated proteins to form autophagosome. The mature autophagosome will dock and fuse with lysosome leading to autophagy, the product of which is used for nutrient recycling. The macronutrients and micronutrients involved in the maintenance of the functions of each organelle are shown in orange boxes. These nutrients play important roles in both the structure of the organelles (such as in cell membrane and DNA stabilization) and the essential physiological processes required to maintain their functions (such as in Krebs cycle, one-carbon cycle, and epigenetic modifications) (Straszewski-Chavez et al., 2005; Kang et al., 2011; Bravo-Sagua et al., 2013; Holland et al., 2017; Rogers et al., 2017; Nakashima et al., 2019a, 2020; Raguema et al., 2020; Zhang et al., 2020).

**FIGURE 2:**
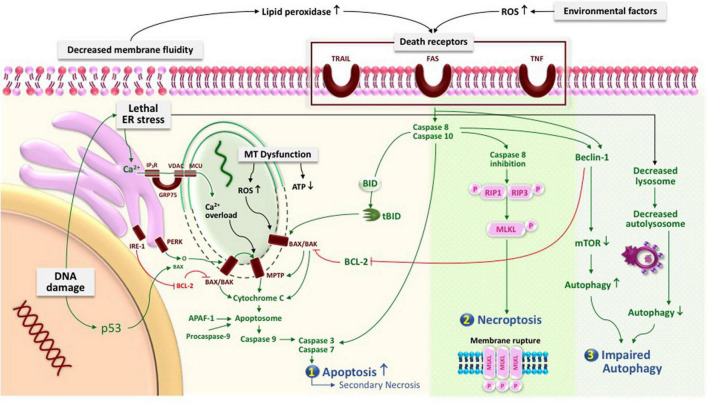
Inappropriate cell death pathways in a trophoblast cell in preeclampsia. Damaged organelles (DNA damage, lethal ER stress, mitochondrial dysfunction, and cell membrane) in trophoblast cell in preeclampsia results in inappropriate cell death: (1) apoptosis, (2) necroptosis, and (3) autophagy.

Figure 2 shows the inappropriate cell death pathways in a trophoblast cell in preeclampsia. Damaged organelles (DNA damage, lethal ER stress, mitochondrial dysfunction, and cell membrane) in trophoblast cells in preeclampsia result in inappropriate cell death: (1) apoptosis, (2) necroptosis, and (3) autophagy. Increased apoptosis is observed in the case of preeclampsia. DNA damage could directly trigger P53 to activate proapoptotic pathways as well as trigger ER–mitochondria cross-talk inducing excessive apoptosis. Excessive ER stress also activates proapoptotic signaling such as IRE-1 and PERK, leading to Ca^2+^ overload in mitochondria. In normal conditions, the presence of calcium could trigger biogenesis and the TCA cycle; however, in preeclampsia, there is excessive ROS and inhibition of biogenesis rate causing mitochondrial dysfunction as well as low ATP production. Both the Ca^2+^ overload from the ER stress and the mitochondrial dysfunction itself will release cytochrome C to induce apoptosome, leading to excessive apoptosis process. However, if apoptotic cells are not scavenged as the apoptotic rate is far beyond normal, they will undergo lytic and inflammatory phases, leading to plasma membrane rupture and thus inducing secondary necrosis. In addition, the excessive lipid peroxidase from decreased membrane fluidity and high ROS from environmental factors could activate the death receptors, resulting in the increase of apoptosis *via* extrinsic pathways by activation of caspase 8 and 10. In this progress, caspase 8 could be inactivated, which enables receptor-interacting protein (RIP)/RIP3 necrosome assembly and triggers MLKL phosphorylation leading to membrane ruptures and necroptosis. On the other hand, autophagy occurs as a result of hypoxia or starvation in preeclampsia where death receptors in the plasma membrane are triggered, leading to activation of Beclin-1, thus enhancing autophagy rate. In contrast, severe preeclampsia results in lethal ER stress where it causes a decreased autolysosome resulting in a decrease in autophagy rate (Powell, 2000; Beyersmann and Haase, 2001; Klug, 2010; Pacheco et al., 2014; Hannan et al., 2017; Lee et al., 2019; Dewi et al., 2020; Guo et al., 2020).

Table 5 is a summary of the physiological functions, pathogenesis in preeclampsia, and the roles of the related nutrition for each component of the trophoblast cell.

**TABLE 5:** Summary table of the role of each cellular health component.

Cellular health components	Physiological functions in placenta	Pathogenesis in preeclampsia	Roles of nutrition
Cell membrane	• Maternal fetal nutrient transport.	•↓ Membrane fluidity	• LCPUFA, particularly DHA.
	• Cellular development.	• Alteration in membrane order.	• Vitamin E.
	• Immune response.	•↓ Nutrient transport.	• Choline.
			• Zinc.
			• Folate.
Mitochondria	• Main source of cellular energy (ATP).	• Altered mitochondrial life cycle.	• Vitamin B complex (B1, B2, B3, B5, B7, B12).
		•↑ Oxidative stress.	• Vitamin C.
		•↑ Mitochondrial damage.	• Vitamin E.
			• Selenium.
			• Zinc.
DNA and epigenetics	• Regulation of gene expression	• Upregulation and downregulation of genes.	• Folate.
		• Hypermethylation and hypomethylation.	• Zinc.
		• miRNAs: miR-210, miR-155, miR-200b.	• Vitamin A.
			• Vitamin B1, B2, B3, B6, B7, B12.
			• Vitamin C.
			• Vitamin D.
			• Vitamin E.
			• Vitamin K.
			• Choline, Betaine.
			• LCPUFA (DHA).
Endoplasmic reticulum	• Site of protein synthesis and folding.	•↑ ER Stress.	• Fatty acid.
	Lipid synthesis.	•↑ Unfolded protein response.	• Obesity.
		•↓ ER-phagy, aggrephagy.	• Calcium.
			• Glucose.
Environmental contaminants		• Persistent organic pollutions (DDT, PCB).	
		• Water pollution (PCE, TCE).	
		• Air pollution (PM2.5, NO_2_).	

## Conclusion

Trophoblasts rely on the integrity of their cellular structure and the physiological mechanism in order to perform all of its functions, especially during placentation. Disruptions in their cellular structures may potentially result in alterations of their cell death mechanisms, resulting in poor invasion and differentiation. Preeclampsia is associated with altered placental cellular wellness caused by intrinsic damages, caused by the dysfunctions of plasma membrane, mitochondrial dysfunction, endoplasmic reticulum stress, changes in DNA methylation, and expression of specific miRNAs, or extrinsic factors due to environmental contaminants. It is important to note that all of these components were interrelated in their functions. Disruptions in their integrity, structure, and functions would result in inappropriate cellular death, hence poor invasion of trophoblasts.

Maternal lifestyle, diet, and the environment may result in modification of placental structure and function. Lifestyle modification including healthy dietary intake, consisting of adequate macronutrients and micronutrients, has the potential to improve cellular wellness, thus preventing preeclampsia. Future studies should focus on providing quantitative evidence on how these nutritional adequacy and supplementation might lead to better cellular structure and functions of trophoblast.

## Author Contributions

AL and RH wrote the first draft. RI and NW edited the manuscript and obtained funding. All authors contributed to the article and approved the submitted version.

## Conflict of Interest

The authors declare that the research was conducted in the absence of any commercial or financial relationships that could be construed as a potential conflict of interest.

## Publisher’s Note

All claims expressed in this article are solely those of the authors and do not necessarily represent those of their affiliated organizations, or those of the publisher, the editors and the reviewers. Any product that may be evaluated in this article, or claim that may be made by its manufacturer, is not guaranteed or endorsed by the publisher.

## Abbreviations

Acetyl-CoA: acetyl coenzyme A
ADAM12: a disintegrin and metalloproteinase-12
AMPK: adenosine monophosphate-activated protein kinase
ARHI: aplasia Ras homolog member I
ATF: activating transcription factor
ATG: autophagy related protein
ATP: adenosine triphosphate
Bax: Bcl-2-associated X
Bcl-2: B-cell lymphoma 2
BCRP: breast cancer resistance protein
BHLNE40: basic helix-loop-helix family member E40
BMP2: bone morphogenetic protein 2
CAT: catalase
CDH11: cadherin 11
Cdkn1c: cyclin dependent kinase inhibitor 1c
CHOP: CCAAT-enhancer-binding protein homologous protein
CPM: confined placental mosaicism
CYP11B2: cytochrome P450 family 11 subfamily B member 2
DDT: dichlorodiphenyltrichloroethane
DHA: docosahexaenoic acid
DNA: deoxyribonucleic acid
DNMT: DNA-methyltransferase
DNMT3L: DNA methyltransferase 3 like
Dsba-L: disulfide-bond A oxidoreductase-like
eNOS: endothelial nitric oxide synthase
ER: endoplasmic reticulum
ERAD: ER-associated proteasomal degradation
ERAP1: endoplasmic reticulum aminopeptidase
ETC: electron transport chain
EVT: extravillous trophoblast
FasL: Fas ligand
FATPs: fatty acid transport proteins
FGF9: fibroblast growth factor 9
FIP200: FAK family kinase-interacting protein of 200 kDa
FIS1: mitochondrial fission 1 protein
FN1: fbronectin 1
FOXO1: forkhead box O1
GATA4: GATA binding protein 4
GLUTs: glucose transporters
GPC: glycerophosphocholine
Grb10: growth factor receptor bound protein 10
H3K9: histone H3 lysine 9
HCH: hexachlorocyclohexane
HDAC6: histone deacetylase 6
HIF1: hypoxia-inducible factor 1
HLA-G: human leukocyte antigen
IGF-1: insulin-like growth factor-1
IGF2: insulin-like growth factor-2
IL: interleukin
INHBA: inhibin subunit beta A
IP3R: inositol triphosphate receptor
IRE1: inositol-requiring transmembrane kinase/endonuclease 1
IRE1 α: inositol-requiring transmembrane kinase/endoribonuclease 1 α
ITGA5: integrin 5 alpha
KRT7: keratin 7
LC3-PEA: light chain 3 –phosphoethanolamine
LCPUFA: long-chain polyunsaturated fatty acids
LDH: lactate dehydrogenase
LDL: low-density lipoprotein
LINE-1: long interspersed elements-1
lncRNA: long non-coding RNAs
MALAT1: metastasis-associated lung adenocarcinoma transcript 1
MAM: mitochondria-associated membranes
MBL: mannose-binding lectin
MCAM: melanoma cell adhesion molecule
MCU: mitochondrial calcium uniporter
MEG3: maternally expressed 3
MFN1: mitofusin 1
miRNA: microRNAs
MLKL: mixed lineage kinase domain-like
MMP: matrix metalloproteinase
mtDNA: mitochondrial DNA
MTHFR: methylenetetrahydrofolate reductase
MTOC: microtubule organizing center
mTORC1: mechanistic target of rapamycin complex 1
MTR: methionine synthase
MTRR: methionine synthase reductase
NK: natural killer
NO: nitric oxide
NO_2_: nitrogen dioxide
NRF-1: nuclear respiratory factor 1
OPA1: optic atrophy-1
OXPHOS: oxidative phosphorylation
P2RX4: purinergic receptor P2 × 4
p53: protein P53
PCB: polychlorinated biphenyl
PCE: perchloroethylene/tetrachloroethylene
PDGFB: platelet-derived growth factor subunit B
Peg1: paternally expressed gene
PERK: protein kinase RNA-like endoplasmic reticulum kinase
PGC-1 α: peroxisome proliferator-activated receptor-gamma coactivator (PGC)-1alpha
PHLDA2: pleckstrin homology like domain family A member 2
PIGF: placental growth factor
PKC β: protein kinase C isoform β
PKR: protein kinase R
PLGF: placental growth factor
PM2.5: particulate matter 2.5
PON-1: paraoxonase-1
POP: persistent organic pollutants
RB1CC1: Rb1 inducible coiled-coil 1
RIP: receptor-interacting protein
RNA: ribonucleic acid
ROS: reactive oxygen species
RUNX3: Runx family transcription factor 3
RyR: ryanodine receptor
SERCA: sarcoendoplasmic reticulum (SR) calcium transport ATPase
SERPINE1: serine protease inhibitor clade E member 1
sFlt-1: Fms-like tyrosine kinase 1
SMAD3: Smad family member 3
SNAs: syncytial nuclear aggregates
SNPs: single-nucleotide polymorphisms
SOD: superoxide dismutase
SPRY4-IT1: Spry4 intronic transcript 1
STAT: signal transducer and activator of transcription
STOX1: storkhead box 1
TCA: tricarboxylic acid
TCE: trichloroethylene
TFAM: transcription factor A, mitochondrial
TFEB: transcriptional factor EB
TGF- β: transforming growth factor- B
TGIF1: Tgfb-induced factor homeobox 1
TIMP3: timp metallopeptidase inhibitor 3
TLR9: toll-like receptor 9
TNF- α: tumor necrosis factor alpha
TNF-Rs: tumor necrosis factor receptors
TNFSF13B: TNF superfamily member 13b
TRAIL: tumor necrosis factor-related apoptosis-inducing ligand
TRAIL-R: TRAIL receptor
ULK: Unc-51-like kinase
UPR: unfolded protein response
VDAC: voltage-dependent anion channels
VEGF: vascular endothelial growth factor
WNT2: wingless-type MMTV integration site family, member 2
ZAC1: *zinc-finger protein which regulates apoptosis and cell-cycle arrest 1*
ZFP57: zinc finger protein 57.
